# Backbone ^1^H, ^15^N and ^13^C resonance assignments of the 27kDa fluorescent protein mCherry

**DOI:** 10.1007/s12104-023-10149-z

**Published:** 2023-09-09

**Authors:** Marco Sette, Laura Anne Johnson, Ralph Jimenez, Frans A.A. Mulder

**Affiliations:** 1 Department of Chemical Sciences and Technology, University of Rome “Tor Vergata”, Rome, Italy; 2 CSPBAT Laboratory, Sorbonne Paris Cité, University of Paris 13, UMR 7244, CNRS, Bobigny, France; 3 Department of Biochemistry, University of Colorado, 596 UCB, Boulder, CO 80309 USA; 4 JILA, University of Colorado, and NIST, Boulder, CO 80309 USA; 5 Department of Chemistry, University of Colorado, 215 UCB, Boulder, CO 80309 USA; 6 Interdisciplinary Nanoscience Center iNANO and Department of Chemistry, University of Aarhus, Aarhus, 8000 Denmark; 7 Institute of Biochemistry, Johannes Kepler Universität Linz, Linz, 4040 Austria

**Keywords:** mCherry, Red fluorescent protein, Protein engineering, Photostability, Chemical shift

## Abstract

mCherry is one of the most successfully applied monomeric red fluorescent proteins (RFPs) for in vivo and in vitro imaging. However, questions pertaining to the photostability of the RFPs remain and rational further engineering of their photostability requires information about the fluorescence quenching mechanism in solution. To this end, NMR spectroscopic investigations might be helpful, and we present the near-complete backbone NMR chemical shift assignment to aid in this pursuit.

## Biotechnological context

Monomeric Red Fluorescent Proteins (mRFPs) are widely used as genetically encodable tags for studying cellular processes. Their distinctive fluorescence results from the chemical rearrangement of amino acids, giving rise to the formation of an acylimine, further modified in different mRFPs. The DsRed precursor (λ_exc_^max^ = 558nm, λ_em_^max^ = 583nm) has the disadvantage of being tetrameric and having low photostability and a slow folding rate. For these reasons, several monomeric variants have been engineered to improve performance in terms of photostability and structural stability and to cover a different range of wavelengths. By using random mutagenesis (Shaner et al. 2004) the series of mFruits was obtained, which comprises mCherry (λ_exc_^max^ = 587nm, λ_em_^max^ = 610nm), mStrawberry (λ_exc_^max^ = 574nm, λ_em_^max^ = 596nm), and mOrange (λ_exc_^max^ = 548nm, λ_em_^max^ = 562nm). Crystal structures of these proteins have been obtained (Shu et al. 2006) and these all show the canonical β-barrel structure harboring an α-helix comprising the residues involved in the formation of the chromophore. The β-barrel in the monomeric mCherry variant (PDB code 2h5q) comprises 11 strands with the chromophore formed by the contiguous residues Methionine-Tyrosine-Glycine (collectively referred to as position 66 in the PDB entry and in our sequence numbering). We report here the near-complete assignment of backbone NMR chemical shifts for mCherry. The N-terminus (residues − 4 to 3) and C-terminus (residues 224–231) are not present in the crystallographic structure and are dynamically disordered. The NMR assignments presented here will be used to address the photostability of the mFruits.

## Method and experiments

### Protein expression and purification

A pET11a plasmid containing the gene of mCherry from *Discosona sp*. (Uniprot code Q5S3G8) containing an N-terminal poly-His tag MGHHHHHHG was transformed in BL21(DE3) *E. coli* cells. A single colony was picked and grown overnight at 37°C in Luria-Bertani media, then spun down and resuspended in 50 mL M9 media to grow overnight. After spinning down, 5 parallel growth flasks were prepared; in each, 4 mL of culture was added to 200 mL M9 medium containing the relevant isotopes (1 gr/L NH_4_Cl and 3 gr/L ^13^C_6_-D-glucose). After growing the cells at 37°C for 4h, 0.001M IPTG was added, and the temperature lowered to room temperature (26°C). Expression was stopped after 24h and the protein pellet was purified on Ni-NTA columns, dialyzed in 50mM Tris pH8.0, 2mM DTT, 0.5mM EDTA and then dialyzed in 20mM Tris, 50mM NaCl pH 7.0. Validation of correct expression of the full-length protein was obtained by ESI-TOF mass spectrometry from which a labelling efficiency of 97% was obtained. The protein used for NMR analysis was inclusive of the poly-His tag.

### NMR spectroscopy

The NMR sample consisted of 1.5 mM U-^15^N,^13^C protein dissolved in 20 mM Tris-HCl, 50 mM NaCl, pH 7.0, 5% D_2_O and spectra were acquired at 303K with a Bruker Avance III HD spectrometer operating at 950MHz proton frequency, equipped with z-axis gradients and a cryogenic probe head (TCI). The following experiments were collected using Bruker library pulse sequences: 2D ^15^N-^1^H HSQC, 3D HNCO, HN(CA)CO, HNCACB, HN(CO)CACB, H(NCA)NH, HN(CO)CA, H(NCOCA)NH, iHNCA, using TROSY (Pervushin et al., 1997) and BEST (Lescop et al., 2007) methodology.

Data were processed with NMRPipe (Delaglio et al. 1995) and spectral assignments were made with NMRFAM-Sparky (Lee et al. 2015). Spectral offsets of 0.5*93 Hz were applied to the amide ^15^N and ^1^H dimensions of all TROSY spectra to align them with the correct decoupled chemical shift values of the 2D ^15^N-^1^H HSQC spectrum. Chemical shift referencing followed the IUPAC recommendation of the protein NMR community (Markley et al., 1998).

## Extent of assignments and data deposition

**Fig. 1:**
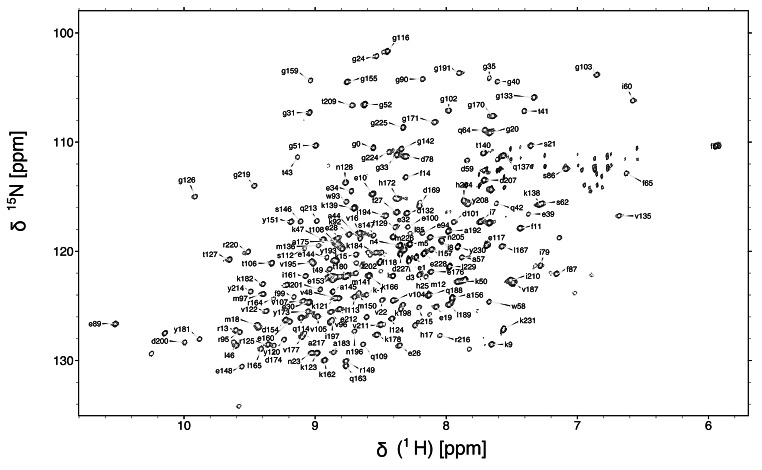
^15^N-^1^H TROSY-HSQC NMR spectrum of mCherry with residue-specific assignments

Despite the presence of more than 236 residues, the ^15^N-^1^H TROSY-HSQC spectrum of mCherry is well resolved (Fig.1), and the sensitivity of 3D BEST-TROSY spectra were adequate for the assignment. Strong signals in the H(NCA)NH and H(NCOCA)NH spectra were helpful for assigning the more dynamic regions, including the termini.

Residue numbering “_Atom_chem_shift.Auth_seq_ID” in the BMRB deposition (ID 51489) follows that reported in the crystal structure (PDB code 2h5q), which runs from Met(-4) to Lys231. The cyclized residues Met-Tyr-Gly are collectively referred to as number 66 and the sequence then continues with Ser69. In the crystal structure, the N-terminal region is disordered and the first residues were not observed. NMR spectra allow the observation of 6 residues in the N-terminal region (from Ser(-2) to Asp3) that were lacking in the electron density map. Their NMR chemical shifts confirm that this region is disordered. Similarly, the last eight residues are not observed in the crystal structure but are assigned in our work and are confirmed disordered. Residues 66–76 containing the chromophore could not be assigned. Also, no assignment was obtained for residues 37, 53–54, 80–83, 130 and 221–222. The completeness of assignments is: ^1^H (199), ^15^N (199), ^13^Cα (211), ^13^Cβ (187), and ^13^C’ (210). Excluding the His-tag, the protein sequence contains 234 amino acids, of which 26 Gly (having no ^13^Cβ) and 12 Pro (lacking ^1^H and yielding no ^15^N assignments in the triple resonance experiments). The extent of completeness for the aforementioned nuclei is then 86%, 86%, 90%, 90%, and 90%, respectively.

**Fig. 2:**
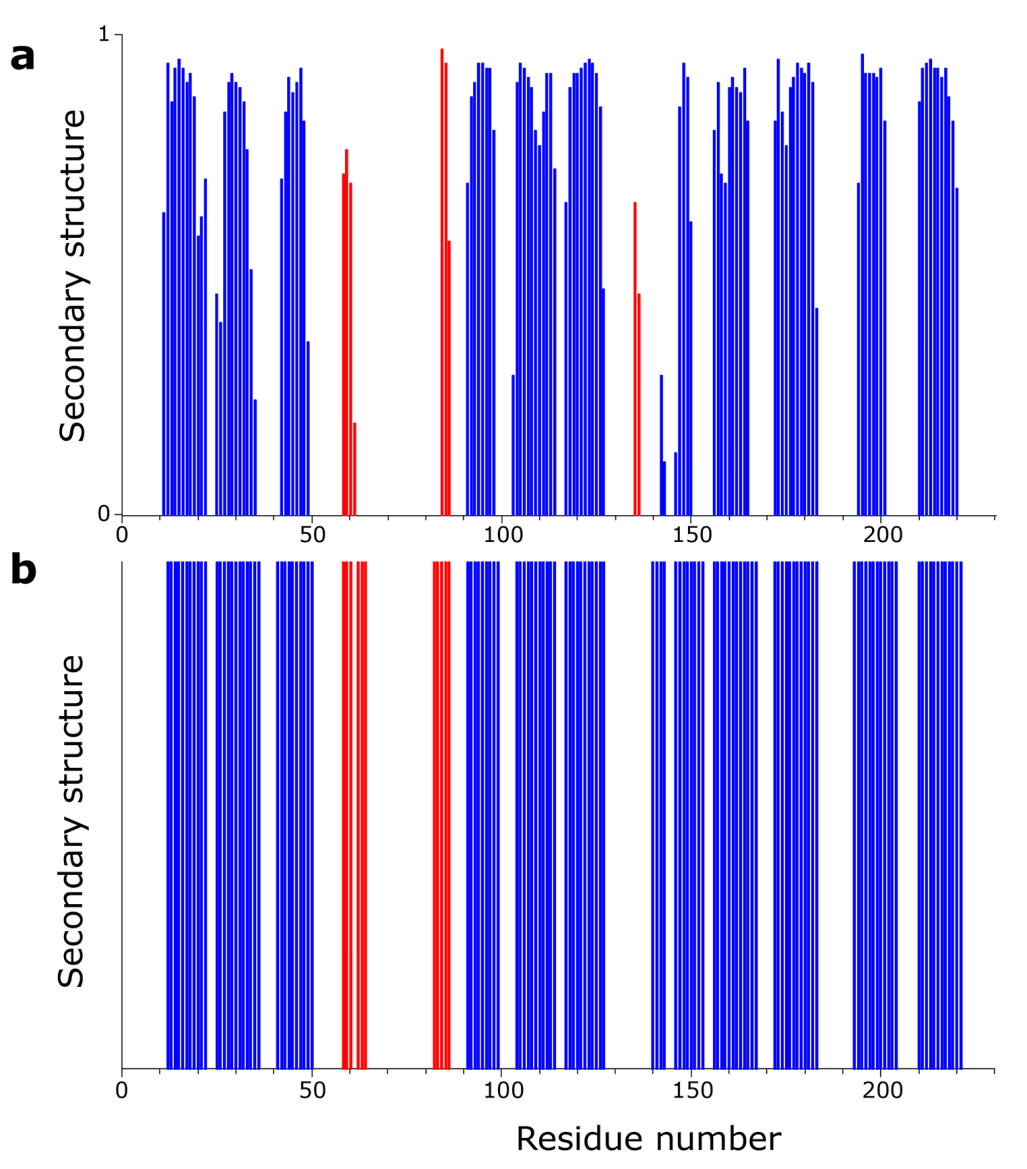
**(a)** Secondary structure of mCherry obtained by using the present NMR chemical shift assignment used as input for the TALOS + software. Blue regions refer to β-strands and red regions to α-helices. **(b)** Secondary structure of mCherry obtained from the crystal structure (PDB code 2h5q) used as input for the STRIDE software. Blue regions refer to β-strands and red regions to α-helices

An intial secondary structure analysis was obtained with the TALOS + software (Shen et al. 2009) and compared with the classification obtained with the STRIDE software (Heinig and Frishman 2004) from the crystallographic structure in Fig.2. Good overall agreement is observed.

**Fig. 3:**
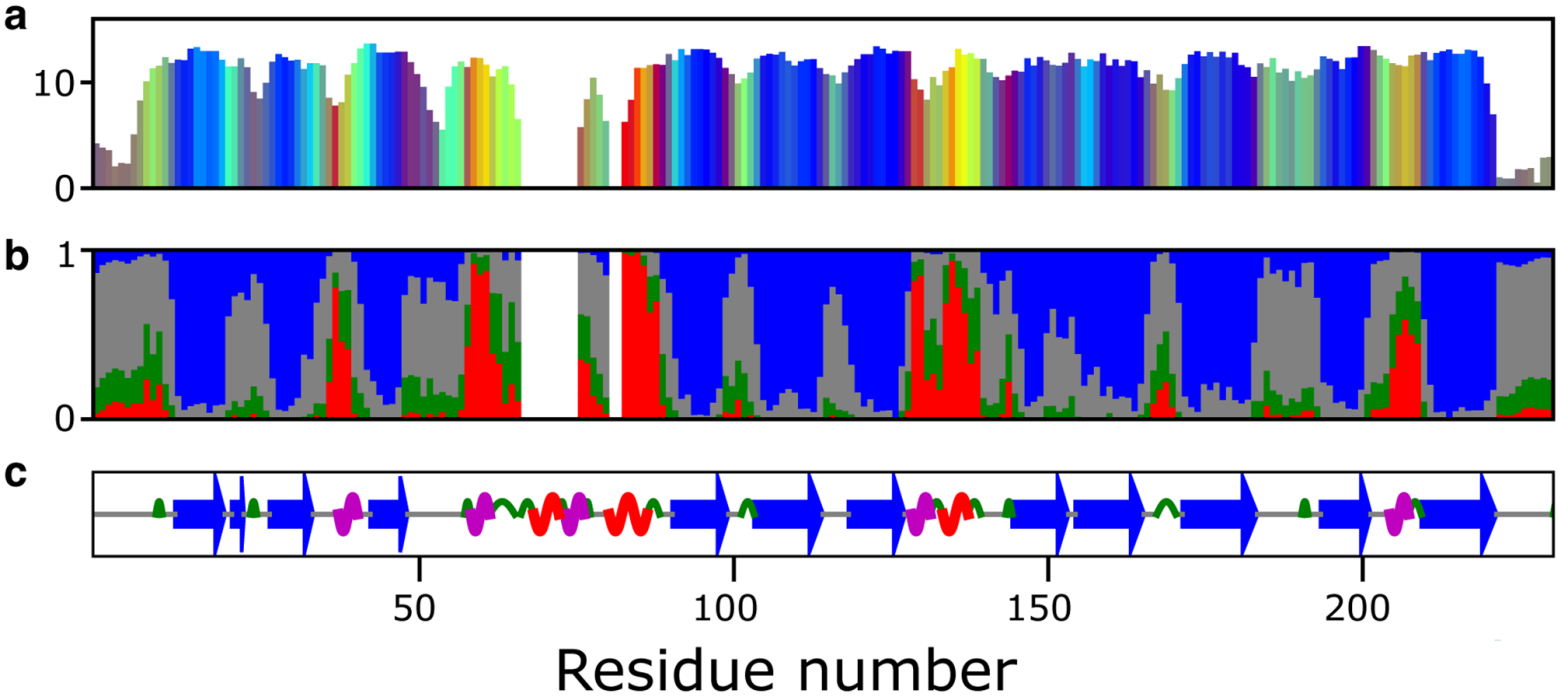
CheSPI analysis for mCherry. **(a)** On the CheSPI color scale, well-formed strands and helices are defined by blue and red colors, respectively, while coil color depends on context; turns are shown in green, and disordered, ‘random coil’, residues are displayed as grey. Hues change from red through orange to yellow at the C-terminal ends of helices and green at the ends of β-strands. For a more comprehensive explanation of the PCA analysis underlying CheSPI colors, the reader is referred to the paper of Nielsen and Mulder (Nielsen and Mulder 2021). **(b)** Stacked bar plot of CheSPI populations of “extended” (blue), “helical” (red), “turn” (green), and “non-folded” (grey), local structures **(c)** CheSPI DSSP-8 assignment. Cartoon of the most confident CheSPI prediction: coil (grey line), turn (green arc), 3_10_-helix (magenta squiggle), α-helix (red squiggle), β-strand (blue arrow)

Although canonical helix and strand conformations are easily detected by programs like TALOS+, the combination of ^1^H, ^15^N, ^13^Cα, ^13^Cβ, and ^13^C’ chemical shifts contains more information about the backbone conformation. It is possible to recover the eight common structural motifs defined as Dictionary of Protein Secondary Structure (DSSP) by the program CheSPI (Nielsen and Mulder 2021). A CheSPI analysis for mCherry is shown in Fig.3. The top panel (a) shows the much richer structural classification, where colors depend on backbone geometry and structural context (see legend). Furthermore, the heights of the bars (CheZOD score) reflects the dynamic information content of the shift information, with a values of 8 marking the border between order and disorder, and values below 3 indicative of ‘random coil’ dynamic averaging. As can be seen in the central panel (b), the canonical secondary structure elements are well retrieved, but some regions diverge from this. The bottom panel (c) shows a summary in which also coil (grey line), turn (green arc), and 3_10_-helices (magenta squiggle) are identified, in addition to α-helix (red squiggle) and β-strand (blue arrow).

## Data Availability

The datasets generated during and/or analysed during the current study are available from the corresponding author on reasonable request. The chemical shifts have been deposited with the BioMagResBank (https://bmrb.io/) and are available under entry number 51489.
